# Adding new experimental arms to randomised clinical trials: Impact on error rates

**DOI:** 10.1177/1740774520904346

**Published:** 2020-02-17

**Authors:** Babak Choodari-Oskooei, Daniel J Bratton, Melissa R Gannon, Angela M Meade, Matthew R Sydes, Mahesh KB Parmar

**Affiliations:** 1 MRC Clinical Trials Unit at UCL, Institute of Clinical Trials and Methodology, University College London, London, UK; 2 Clinical Statistics, GlaxoSmithKline, Uxbridge, UK; 3 Department of Health Services Research and Policy, London School of Hygiene & Tropical Medicine, London, UK

**Keywords:** Platform trials, adaptive trial designs, familywise type I error rate, pairwise error rate, STAMPEDE trial, multi-arm multi-stage, MAMS, survival time

## Abstract

**Background:**

Experimental treatments pass through various stages of development. If a treatment passes through early-phase experiments, the investigators may want to assess it in a late-phase randomised controlled trial. An efficient way to do this is adding it as a new research arm to an ongoing trial while the existing research arms continue, a so-called multi-arm platform trial. The familywise type I error rate is often a key quantity of interest in any multi-arm platform trial. We set out to clarify how it should be calculated when new arms are added to a trial some time after it has started.

**Methods:**

We show how the familywise type I error rate, any-pair and all-pairs powers can be calculated when a new arm is added to a platform trial. We extend the Dunnett probability and derive analytical formulae for the correlation between the test statistics of the existing pairwise comparison and that of the newly added arm. We also verify our analytical derivation via simulations.

**Results:**

Our results indicate that the familywise type I error rate depends on the shared control arm information (i.e. individuals in continuous and binary outcomes and primary outcome events in time-to-event outcomes) from the common control arm patients and the allocation ratio. The familywise type I error rate is driven more by the number of pairwise comparisons and the corresponding (pairwise) type I error rates than by the timing of the addition of the new arms. The familywise type I error rate can be estimated using Šidák’s correction if the correlation between the test statistics of pairwise comparisons is less than 0.30.

**Conclusions:**

The findings we present in this article can be used to design trials with pre-planned deferred arms or to add new pairwise comparisons within an ongoing platform trial where control of the pairwise error rate or familywise type I error rate (for a subset of pairwise comparisons) is required.

## Introduction

Many recent developments in clinical trials are aimed at speeding up research by making better use of resources. Phase III clinical trials can take several years to complete in many disease areas, requiring considerable resources. During this time, a promising new treatment which needs to be tested may emerge. The practical advantages of incorporating such a new experimental arm into an existing trial protocol have been clearly described before,^1–4^ not least because it obviates the often lengthy process of initiating and launching a new trial which may compete for patients with the existing one. One trial using this approach is the STAMPEDE trial^5^ in men with high-risk prostate cancer. STAMPEDE is a multi-arm multi-stage (MAMS) platform trial that was initiated with one common control arm and five experimental arms assessed over four stages. Five new experimental arms have been added since its conception^1^– see next section for further design details. This was done within the paradigm of a ‘platform’ that has a single master protocol in which multiple treatments are evaluated over time. It offers flexible features such as early stopping of accrual to treatments for lack-of-benefit or adding new research treatments to be tested during the course of a trial. There might also be scenarios when at the design stage of a new trial another experimental arm is planned to be added after the start of the trial, that is, a planned addition. An example of this scenario is the RAMPART trial in renal cancer – see “Results” section and online Supplemental Appendix for design details. In some platform designs, however, the addition of the new experimental arm would be intended but not specially planned at the start of the platform, that is, unplanned, and is opportunistic at a later stage. In other words, in the planned scenario, the addition of a new research arm at a later stage is clearly foreseen at the design stage, whereas in the unplanned scenario the opportunity or need to assess a further comparison becomes apparent during the trial.

The type I error rate is one of the key quantities in the design of any clinical trial. Two measures of type I error in a multi-arm trial are the pairwise type I error rate (PWER) and familywise type I error rate (FWER). The PWER is the probability of incorrectly rejecting the null hypothesis for the primary outcome of a particular experimental arm at the end of the trial, regardless of other experimental arms in the trial. The FWER is the probability of incorrectly rejecting the null hypothesis for the primary outcome for at least one of the experimental arms from a set of comparisons in a multi-arm trial. It gives the type I error rate for a set of pairwise comparisons of the experimental arms with the control arm. In trials with multiple experimental arms, the maximum possible FWER often needs to be calculated and known – see the review by Wason et al.^6^ for details. In some multi-arm trials, this maximum value needs to be controlled at a predefined level. This is called a *strongly* controlled FWER as it covers all eventualities, that is, all possible hypotheses.^7^ Dunnett^8^ developed an analytical formula to calculate the FWER in multi-arm trials when all the pairwise comparisons of experimental arms against the control arm start and conclude at the same time. However, it has been unclear how to calculate the FWER when new experimental arms are added during the course of a trial.

The purpose of this article is threefold. First, we describe how the FWER, disjunctive (any-pair) and conjunctive (all-pairs) powers – see “Methods” section for their definitions – can be calculated when a new experimental arm is added during the course of an existing trial with continuous, binary, and time-to-event outcomes. Second, we describe how the FWER can be *strongly* controlled at a prespecified level for a set of pairwise comparisons in both planned (i.e. the added arm is planned at the design stage) and unplanned (e.g. such as platform designs) scenarios. Third, we explain how the decision to control the PWER or the FWER in a particular design involves a subtle balancing of both practical and statistical considerations.^9^ This article outlines these issues and provides guidance on whether to emphasise the PWER or the FWER in different design scenarios when adding a new experimental arm.

The structure of the article is as follows. In the next section, the design of the STAMPEDE platform trial is presented. In “Methods” section, we explain how the FWER, disjunctive, and conjunctive powers are computed when a new experimental arm is added to an ongoing trial. In “Results” section, we present the outcome of our simulations to verify our analytical derivation. We also show two applications in both planned (i.e. RAMPART trial in renal cancer) and platform design (i.e. STAMPEDE trial) settings. We then propose strategies that can be applied to *strongly* control the FWER when adding new experimental arms to an ongoing platform trial in scenarios where such a control is required. Finally, we discuss our findings.

## An example: STAMPEDE trial

STAMPEDE^1^ is a multi-arm multi-stage (MAMS) platform trial for men with prostate cancer at high risk of recurrence who are starting long-term androgen deprivation therapy. In a four-stage design, five experimental arms with treatment approaches previously shown to be safe were compared with a control arm regimen. In this trial, the primary analysis was carried out at the end of stage 4, with overall survival as the primary outcome. Stages 1 to 3 used an intermediate outcome measure of failure-free survival. As a result, the corresponding hypotheses at interim stages played a subsidiary role – that is, used for lack-of-benefit analysis on an intermediate outcome, not for making claims of efficacy. We, therefore, focus here on the primary hypotheses on overall survival at the final stage – for designs with both lack-of-benefit and efficacy stopping boundaries see the articles by Blenkinsop et al.^10^ and Blenkinsop and Choodari-Oskooei.^11^

Recruitment to the original arms began late in 2005 and was completed early in 2013. The design parameters for the primary outcome at the final stage were a (one-sided) pairwise significance level of 0.025, power of 0.90, and the target hazard ratio of 0.75 on overall survival which required 401 control arm deaths (i.e. events on overall survival). An allocation ratio of A=0.5 was used for the original comparisons so that, over the long term, one patient was allocated to each experimental arm for every two patients allocated to control. Because distinct hypotheses were being tested in each of the five experimental arms, the emphasis in the design for STAMPEDE was on the pairwise comparisons of each experimental arm against control, with emphasis on the strong control of the PWER. Out of the initial five experimental arms, only three of them continued to recruit through to their final stage. Recruitment to the other two arms stopped at the second interim look due to lack of sufficient activity.

**Figure 1.**
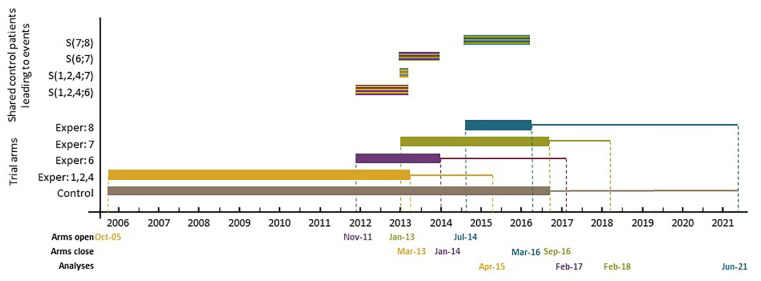
Schematic representation of the control and experimental arm timelines in the STAMPEDE trial. Bottom section: the thick horizontal bars represent the accrual period, and the following solid lines represent the follow-up period. Top section: the striped bars represent the period when the recruited control arm patients overlap during this period between different pairwise comparisons. The colours of the stripes represent the colours of each pairwise comparison. For example, the striped bar that is labelled as S(1,2,4;6) represents the period when the recruited control arm patients are shared between the original pairwise comparisons 1,2, and 4 and the sixth newly added comparison during this period.

Since November 2011, five new experimental arms have been added to the original design. Figure 1 presents the timelines for different arms, including three of those added later. Note that patients allocated to a new experimental arm are only compared with patients randomised to the control arm contemporaneously, and recruitment to the new experimental arm(s) continues for as long as is required. Therefore, the analysis and reporting of the new comparisons will be later than for the original comparisons. Figure 1 also shows the recruitment periods when the pairwise comparisons of the newly added experimental arms with the control overlap with each other as well as with those of the original comparisons – see the top section of Figure 1.

## Methods

In this section, we first present the formulae for the correlation of the two test statistics when one of the comparisons is added later in trials with continuous, binary, and survival outcomes. We then describe how Dunnett’s test can be extended to compute the FWER, as well as conjunctive and disjunctive powers when a new arm is added mid-course of a two-arm trial.

### Type I error rates when adding a new arm

In a two-arm trial, the primary comparison is between the control group (*C*) and the experimental treatment (*E*). The parameter θ represents the difference in the outcome measure between the two groups. In the notation of this article, the control group is always identified with subscript 0. For continuous outcomes, θ could be the difference in the means of the two groups $\mu_{1}-\mu_{0}$; for binary data difference in the proportions $p_{1}-p_{0}$; and for survival data a log hazard ratio (logHR).

The efficient score statistic for θ (based on the available data and calculated under the null hypothesis that θ=0) is represented by *S* with *V* being Fisher’s (observed) information about θ contained in *S*. Conditionally on the value of *V*, in large samples (which is the underlying assumption throughout this article), *S* follows the normal distribution with mean θV and variance *V*, that is, S~N(θV,V). In the survival case, *S* and *V* are the logrank test statistic and its null variance, respectively.

In practice, the progress of a trial can be assessed in terms of ‘information time’*t* because it measures how far through the trial we are.^12^ In the case of continuous and binary outcomes, *t* is defined as the total number of individuals accrued so far divided by the total sample size. In survival outcomes, it is defined as the total number of events occurred so far divided by the total number of events required by the planned end of the trial.^12^ In all cases t=0 and t=1 correspond to the beginning and end of the trial, respectively. In continuous, binary, and survival outcome data, *S* has independent and normally distributed increment structure. This means that at information times $t_{1},t_{2},\ldots ,t_{j}$, the increments $S(t_{1}),S(t_{2})-S(t_{1}),\ldots ,S(t_{j})-S(t_{j-1})$ are independently and normally distributed.

Furthermore, the *Z*-test statistic can be expressed in terms of the efficient score statistic *S* and Fisher’s information as $Z=S/\sqrt{V}$. The *Z*-test statistic is (approximately) normally distributed $Z\sim N(\theta \sqrt{V},1)$ and has the same independent increment property as that of *S*. For example, in trials with continuous outcomes, where the aim is to test that the outcome of $n_{1}$ individuals in experimental treatment $E_{1}$ is on average smaller (here smaller means better, for example, blood pressure) than that of $n_{0}$ individuals in control group (*C*), the null hypothesis $H_{0}^{1}:\mu_{1}\geq \mu_{0}$ is tested against the (one-sided) alternative hypothesis $H_{1}^{1}:\mu_{1}<\mu_{0}$. In this case, the type I error rate is a predefined value $\alpha_{1}=\Phi (z_{\alpha_{1}})$ where Φ(.) is the normal probability distribution function. Denote $Z_{1}$ the standardised test statistics for $E_{1}$ versus control. Under $H_{0}$, the distribution of the *z*-test statistics is standard normal, N(0,1). Table 1 presents the test statistics for continuous, binary, and survival outcomes with the corresponding Fisher’s (observed) information.

**Table 1.** Treatment effects, test statistics, expected information, and correlation between the test statistics of pairwise comparisons in trials with continuous, binary, and survival outcomes, with common allocation ratio (*A*).

Outcome	Treatment effect (θ)	Test statistics (*Z*)	Fisher’s information (*V*)	Correlation between two comparisons	
				Complete overlap ($\rho_{12}$)	Partial overlap ($\rho_{12}^{*}$)
Continuous	$\theta_{c}=\mu_{1}-\mu_{0}$	$Z_{1}=\theta_{c}\sqrt{V_{c}}$	$V_{c}={\left(\frac{\sigma_{0}^{2}}{n_{0}}+\frac{\sigma_{1}^{2}}{An_{0}}\right)}^{-1}$	$\frac{A}{A+1}$	$\frac{A}{A+1}.\frac{n_{0,12}}{n_{0}}$
Binary{	$\theta_{b_{1}}=p_{1}-p_{0}$	$Z_{1}=\theta_{b_{1}}\sqrt{V_{b_{1}}}$	$V_{b_{1}}={\left(\frac{p_{0}(1-p_{0})}{n_{0}}+\frac{p_{1}(1-p_{1})}{An_{0}}\right)}^{-1}$	$\frac{A}{A+1}$	$\frac{A}{A+1}.\frac{n_{0,12}}{n_{0}}$
	$\theta_{b_{2}}=\log\left\{\frac{p_{1}(1-p_{0})}{p_{0}(1-p_{1})}\right\}$	$Z_{1}=\theta_{b_{2}}\sqrt{V_{b_{2}}}$	$V_{b_{2}}={\left(\frac{1}{n_{0}p_{0}(1-p_{0})}+\frac{1}{An_{0}p_{1}(1-p_{1})}\right)}^{-1}$	$\frac{A}{A+1}$	$\frac{A}{A+1}.\frac{n_{0,12}}{n_{0}}$
	$\theta_{b_{3}}=\log\left\{\frac{p_{1}}{p_{0}}\right\}$	$Z_{1}=\theta_{b_{3}}\sqrt{V_{b_{3}}}$	$V_{b_{3}}={\left(\frac{1-p_{0}}{n_{0}p_{0}}+\frac{1-p_{1}}{An_{0}p_{1}}\right)}^{-1}$	$\frac{A}{A+1}$	$\frac{A}{A+1}.\frac{n_{0,12}}{n_{0}}$
Survival	$\theta_{s}=\log(\mathrm{HR})$	$Z_{1}=\theta_{s}\sqrt{V_{s}}$	$V_{s}={\left(d\frac{A}{{\left(1+A\right)}^{2}}\right)}^{-1}$	$\frac{A}{A+1}$	$\frac{A}{A+1}.\frac{e_{0,12}}{e_{0}}$

$\rho_{12}$: correlation when there is complete overlap between pairwise comparisons. $\rho_{12}^{*}$: correlation when only $n_{0,12}(e_{0,12})$ control arm observation (events) overlaps between comparisons 1 and 2. $n_{0,12}(e_{0,12})$, shared observations (events) in control arm; $n_{0}(e_{0})$, total observations (events) in control arm; *d*, all events.

If a different experimental arm, $E_{2}$ is compared with the control treatment *C* in another independent trial, the corresponding null hypothesis is $H_{0}^{2}:\mu_{2}\geq \mu_{0}$ with the type I error rate being similarly defined as $\alpha_{2}$. Magirr et al.^13^ showed that the FWER is maximised under the global null hypothesis, $H_{0}^{G}$, that is, when the mean outcome in each of the experimental arms is equal to that of the control arm, $H_{0}^{G}:\theta_{1}^{0}=\theta_{2}^{0}=0$. In the above scenario, since the two trials are independent, the overall type I error rate (FWER) of the two comparisons, k=1,2, can be calculated using the Šidák^14^ formula

$$\begin{array}{c} \mathrm{FWE}R_{S}=\mathrm{Pr}(\mathrm{reject}\;\mathrm{at}\;\mathrm{least}\;\mathrm{one}\;H_{0}^{k}|H_{0}^{G})\\ =\mathrm{Pr}(\mathrm{reject}\;H_{0}^{1}\;\mathrm{or}\;H_{0}^{2}|H_{0}^{G})\\ =1-\mathrm{Pr}(\mathrm{accept}\;H_{0}^{1}\;\mathrm{and}\;H_{0}^{2}|H_{0}^{G})\\ =1-(1-\alpha_{1})(1-\alpha_{2}) \end{array}$$

When $\alpha_{1}=\alpha_{2}=\alpha$

(1) $$\mathrm{FWE}R_{S}=1-(1-\alpha {)}^{2}$$

where subscript *S* stands for Šidák. If the control arm observations are shared between the two pairwise comparisons, one can replace the term $(1-\alpha {)}^{2}$ in equation (1) to allow for the correlation between the two test statistics $Z_{1}$ and $Z_{2}$, that is, the correlation induced by the shared control arm information. Dunnett^8^ provided an analytical formula to estimate the FWER when all the comparisons start and conclude at the same time, that is, when all control arm observations overlap between different comparisons. In the above scenario, the FWER can be calculated using

(2) $$\mathrm{FWE}R_{D}=1-\Phi_{2}\left(z_{1-\alpha_{1}},z_{1-\alpha_{2}};\rho_{12}\right)$$

where $\Phi_{2}(.;\rho_{12})$ is the standard bivariate normal probability distribution function and $\rho_{12}$ is the correlation between $Z_{1}$ and $Z_{2}$ at the final analysis. With equal allocation ratio $A=A_{1}=A_{2}$ across all experimental arms, $\rho_{12}=A/A+1$, for example, $\rho_{12}=0.5$ when $n_{0}=n_{1}=n_{2}$.^8^

The formula for $\rho_{12}$ can be extended for the scenario when $E_{2}$ is started later than $E_{1}$ and *C* overlaps with both of them, that is, when only some of the control arm observations are shared between the two comparisons. This scenario is quite common in platform trials where new experimental arms can be added to the previous sets of pairwise comparisons, and recruitment to the new experimental and control arms continues until the planned end of that comparison. To achieve this, we make use of the asymptotic properties of the efficient score statistic and the *z*-test statistic. It has been shown that over time, the sequence of *z*-test statistics approximately has an independent and normally distributed increment structure for the estimators of the treatment effects presented in Table 1.^12,15^ This means that at information time t′>t

$$\sqrt{N(t\prime )}Z(t\prime )=\sqrt{N(t)}Z(t)+\sqrt{N(t\prime )-N(t)}Z(t\prime -t)$$

where N(t′) and N(t) are the total sample sizes at information times t′ and *t*. With equal allocation ratio to both experimental arms, if $n_{0,12}$ control arm observations (where $0<n_{0,12}<n_{0}$) are shared between the two comparisons, the correlation between $Z_{1}$ and $Z_{2}$ can be calculated using equation (3) – see online Supplemental Appendix for analytical derivations and more complex formula for the case of unequal allocation ratio between comparisons

(3) $$\rho_{12}^{*}=\frac{A}{A+1}.\frac{n_{0,12}}{n_{0}}$$

Note that the factor $n_{0,12}/n_{0}$ is bounded by [0,1] with the upper bound equal to 1 when $n_{0,12}=n_{0}$ (i.e. when the two comparisons start and finish at the same time), and the lower bound equal to 0 when there is no shared observation in the control arm – in which case, $\mathrm{FWE}R_{D}$ converges to $\mathrm{FWE}R_{S}$.

Our analytical derivation shows that equation (3) applies to both continuous and binary outcome measures. However, in survival outcomes, the ratio $n_{0,12}/n_{0}$ should be replaced with the ratio of the shared events in the control arm, that is, $e_{0,12}/e_{0}$– see Supplemental Appendix for analytical derivations and also the more complicated formula for unequal allocation ratio. Table 1 shows the corresponding formula for $\rho_{12}^{*}$ by the type of outcome measure.

### Power when adding a new arm

The power of a clinical trial is the probability that under a particular target treatment effect $\theta^{1}$, a truly effective treatment is identified at the final analysis. In multi-arm designs, per-pair (pairwise) power (ω)^16^ calculates this probability for a given experimental arm against the control. In multi-arm settings, however, there are other definitions of power that might be of interest – depending on the objective of the trial. In the above setting where there are two comparisons, define the target treatment effects under the alternative hypothesis for each of the comparisons as $\theta_{1}^{1}$ and $\theta_{2}^{1}$, respectively. Disjunctive (any-pair) power is the probability of showing a statistically significant effect under the targeted effects for at least one comparison

$$\begin{array}{c} \Omega_{d}=\mathrm{Pr}(\mathrm{reject}\;\mathrm{at}\;\mathrm{least}\;\mathrm{one}\;H_{0}^{k}|\theta_{1}=\theta_{1}^{1},\theta_{2}=\theta_{2}^{1})\\ =1-\mathrm{Pr}(\mathrm{accept}\;H_{0}^{1}\;\mathrm{and}\;H_{0}^{2}|\theta_{1}=\theta_{1}^{1},\theta_{2}=\theta_{2}^{1}) \end{array}$$

when the two comparisons, k=1,2, are independent, that is, $\rho_{12}=0$, disjunctive power $\left(\Omega_{d}\right)$ is defined as

(4) $$\Omega_{d}=1-(1-\omega_{1})(1-\omega_{2})$$

If $\rho_{12}\neq 0$, then $\Omega_{d}$ is calculated using

(5) $$\Omega_{d}=1-\Phi_{2}(z_{1-\omega_{1}},z_{1-\omega_{2}};\rho_{12})$$

Conjunctive (all-pairs) power is the probability of showing a statistically significant effect under the targeted effects for all comparison pairs. When the two tests are independent, conjunctive power $\left(\Omega_{c}\right)$ is

$$\begin{array}{c} \Omega_{c}=\mathrm{Pr}(\mathrm{reject}\;\mathrm{all}\;H_{0}^{k}|\theta_{1}=\theta_{1}^{1},\theta_{2}=\theta_{2}^{1})\\ =\mathrm{Pr}(\mathrm{reject}\;H_{0}^{1}\;\mathrm{and}\;H_{0}^{2}|\theta_{1}=\theta_{1}^{1},\theta_{2}=\theta_{2}^{1})\\ =\omega_{1}\cdot \omega_{2} \end{array}$$

Given the correlation $\rho_{12}$, then $\Omega_{c}$ is calculated using

(6) $$\Omega_{c}=\Phi_{2}(z_{\omega_{1}},z_{\omega_{2}};\rho_{12})$$

If a new experimental arm is added later on, the corresponding formula for $\rho_{12}^{*}$ in Table 1 can be used to calculate both disjunctive $\left(\Omega_{d}\right)$ and conjunctive $\left(\Omega_{c}\right)$ powers in this scenario.

## Results

In this section, we first show the results of our simulations to explore the validity of equation (3) to estimate $\rho_{12}^{*}$ and to study the impact of the timing of the addition of a new experimental arm on the FWER and different types of power. Because of the censoring, survival outcomes are generally considered the most complex type of outcomes listed in Table 1. We conduct our simulations in this setting. Then, we estimate the correlation structure between the test statistics of different comparisons in the STAMPEDE trial, including the first three of the added arms to the original set of comparisons. Finally, to illustrate the design implications in planned scenarios, we show an application in the design of the RAMPART trial.

### Simulation design

In our simulations, we considered a hypothetical three-arm trial with one control, *C*, and two experimental arms ($E_{1}$ and $E_{2}$). We applied similar design parameters to those in the article by Royston et al.^16^– see Section 2.7.1–taking median survival for the time-to-event outcome of 1 year (hazard $\lambda_{1}=0.693$ in control arm). We generated individual time-to-event patient data from an exponential distribution and estimated the correlation between the test statistics of the two pairwise comparisons $Z_{1}$ and $Z_{2}$ when $E_{2}$ was initiated at different time points after the start of the experimental arm $E_{1}$ and control. Accrual rates were assumed to be uniform throughout (across the platform) and set to 500 patients per unit time for both comparisons.

As in the STAMPEDE trial, the comparison set of patients for the deferred experimental arm $E_{2}$ are the contemporaneously recruited control arm *C* individuals. This means that in our simulations, recruitment to the control arm continued until conclusion of that required for the $E_{2}$ comparison. As for the final stage of STAMPEDE, the design significance level and power were chosen as $\alpha_{i}=0.025$ and $\omega_{i}=0.9$, i=1,2 in all scenarios. The target hazard ratio under the alternative hypothesis in both pairwise comparisons was 0.75. To investigate the FWER under different allocation ratios, we carried out our simulations under three allocation ratios of A={0.5,1,2}. Table 2 presents details of the design parameters, including trial timelines, in each pairwise comparison for different values of *A*. Calculations for Table 2 were done in Stata using the nstage programme.^17^ In simulations, 50,000 replications were generated in each scenario.

**Table 2.** Three different trial designs for each pairwise comparison of experimental arm versus control in a three-arm trial.

Scenario	*A*	$e_{0}$	$n_{0}$	Overall trial period
1	0.5	401	789	2.36
2	1	264	545	2.18
3	2	196	389	2.33

Key: *A*, allocation ratio; $e_{0}$, total control arm events required; $n_{0}$, number of patients accrued to control arm by the end of trial; overall trial period, duration (in time units) up to the final analysis.

Finally, the main aim of our simulation study is to explore the impact of the timing of adding a new experimental arm on the correlation structure and the value of the FWER. For this reason, only one original comparison was included in our simulations. In the following sections and Discussion, we discuss how the FWER can be strongly controlled and address other relevant design issues, when more pairwise comparisons start at the beginning.

### Simulation results

The results are summarised in Tables 3 and 4. Table 3 shows the values of the correlation between the test statistics of the two pairwise comparisons as computed from the corresponding equation for $\rho_{12}^{*}$ in Table 1, by the timing of when the second experimental arm $E_{2}$ was added. We estimated the number of shared control arm events, $e_{0,12}$, via simulation. For each scenario, we also simulated individual patient data under the null hypothesis and computed both test statistics, which were then used to estimate the correlation between them. The results are also included in Table 3, that is, ${\hat{\rho }}_{12}^{*}$. The results indicate that the estimates ${\hat{\rho }}_{12}^{*}$ accord well with the corresponding values obtained from the formula in all experimental conditions – they mostly differed in the third decimal place.

**Table 3.** Estimates and real values of the correlation between the test statistics of the two pairwise comparisons, $Z_{1}$ and $Z_{2}$, by the timing of the addition of experimental arm $E_{2}$.

Time $E_{2}$ started	AllocationRatio=0.5				AllocationRatio=1				AllocationRatio=2			
	Shared control arm events	$\rho_{12}^{*}$	${\hat{\rho }}_{12}^{*}$	FWER	Shared control arm events	$\rho_{12}^{*}$	${\hat{\rho }}_{12}^{*}$	FWER	Shared control arm events	$\rho_{12}^{*}$	${\hat{\rho }}_{12}^{*}$	FWER
0.0	401	0.33	0.33	0.047	264	0.50	0.50	0.045	196	0.66	0.66	0.043
0.2	348	0.29	0.29	0.048	226	0.43	0.43	0.046	170	0.58	0.57	0.044
0.4	298	0.25	0.25	0.048	189	0.36	0.36	0.047	144	0.49	0.49	0.045
0.6	249	0.20	0.20	0.048	155	0.29	0.29	0.048	121	0.41	0.40	0.046
0.8	204	0.17	0.16	0.049	123	0.23	0.23	0.048	98	0.33	0.33	0.047
1.0	161	0.13	0.14	0.049	94	0.18	0.18	0.049	77	0.26	0.26	0.048
1.2	122	0.10	0.10	0.049	67	0.13	0.13	0.049	58	0.20	0.20	0.049
1.4	87	0.07	0.07	0.049	45	0.09	0.09	0.049	41	0.14	0.15	0.049
1.6	57	0.05	0.05	0.049	26	0.05	0.05	0.049	26	0.09	0.08	0.049
1.8	33	0.03	0.03	0.049	12	0.02	0.03	0.049	15	0.05	0.07	0.049
2.0	14	0.01	0.02	0.050	3	0.01	0.01	0.050	6	0.02	0.04	0.049

FWER: familywise type I error rate.

The values for $\rho_{12}^{*}$ are calculated from equation (3). The estimates ${\hat{\rho }}_{12}^{*}$ are obtained from simulating individual patient data. The number of trial-level replicates is 50,000 in all experimental conditions.

**Table 4.** Disjunctive ($\Omega_{d}$) and conjunctive ($\Omega_{c}$) powers by the timing of the addition of the second arm and the correlation between the test statistics of the two pairwise comparisons.

Time $E_{2}$ started	AllocationRatio=0.5			AllocationRatio=1			AllocationRatio=2		
	$\rho_{12}^{*}$	$\Omega_{d}$	$\Omega_{c}$	$\rho_{12}^{*}$	$\Omega_{d}$	$\Omega_{c}$	$\rho_{12}^{*}$	$\Omega_{d}$	$\Omega_{c}$
0.0	0.33	0.977	0.823	0.50	0.968	0.833	0.66	0.956	0.844
0.2	0.29	0.979	0.821	0.43	0.972	0.828	0.58	0.963	0.837
0.4	0.25	0.980	0.819	0.36	0.975	0.825	0.49	0.968	0.832
0.6	0.20	0.983	0.817	0.29	0.979	0.821	0.41	0.972	0.827
0.8	0.17	0.984	0.816	0.23	0.982	0.819	0.33	0.977	0.823
1.0	0.13	0.986	0.815	0.18	0.984	0.817	0.26	0.980	0.820
1.2	0.10	0.987	0.813	0.13	0.986	0.815	0.20	0.983	0.817
1.4	0.07	0.988	0.812	0.09	0.987	0.813	0.14	0.985	0.815
1.6	0.05	0.988	0.812	0.05	0.988	0.812	0.09	0.987	0.813
1.8	0.03	0.989	0.811	0.02	0.989	0.810	0.05	0.988	0.812
2.0	0.01	0.990	0.810	0.01	0.990	0.810	0.02	0.989	0.810

The results for each allocation ratio indicate that when $E_{2}$ starts later than $E_{1}$ and *C*, the estimates ${\hat{\rho }}_{12}^{*}$ and the FWER are driven by the shared control arm events between the two pairwise comparisons (see Table 3). The higher the number of the shared control arm events, the lower the value of the FWER is because $\rho_{12}^{*}$ is higher. The FWER reaches its maximum when there is no shared information between the two pairwise comparisons at which point equation (1) can be used to calculate the FWER. This is when the two pairwise comparisons are effectively two completely independent trials in one protocol. In this case, Bonferroni correction can also provide a good approximation, that is, $\alpha_{1}+\alpha_{2}=0.05$. The results indicate that even for a correlation of as high as 0.30, the Bonferroni correction provides a good approximation. This correlation threshold corresponds to an overlap (in terms of ‘information time’) of about 60% between the newly added comparison and that of the existing one for an equal allocation ratio (A=1). If more individuals are allocated to the control arm (i.e. A<1), the amount of overlap has to be even higher to achieve this correlation threshold, for example, about 87% when A<1 in our simulations for either experimental arm.

Furthermore, it is evident from our simulations that when more individuals are allocated to the control arm (i.e. A<1), the timing of adding a new experimental arm has very little impact on the value of the FWER. For an equal allocation ratio (A=1), the impact on the FWER is modest, whereas for the uncommon scenario of allocation ratio A>1, the impact is moderate. Therefore, in many multi-arm trials, where often more individuals are allocated to the control arm than each experimental arm, the timing of the addition of a new experimental arm is unlikely to be a major issue.

Finally, Table 4 presents the disjunctive and conjunctive powers in each scenario. The results indicate that the timing of the addition of the new arm has more impact on both types of powers. Nonetheless, the impact is still relatively low – particularly, when the allocation ratio is less than one. However, the degree of overlap affects the two types of powers in opposite directions. While conjunctive power decreases with smaller overlap, disjunctive power increases in such scenario.

### FWER of STAMPEDE when new arms were added

In this section, we calculate the correlation between the test statistics of different pairwise comparisons in STAMPEDE when new arms are added. The newly added therapies look to address different research questions than those of the original comparisons. When the first new experimental arm was added, STAMPEDE had only three experimental arms open to accrual because arms $E_{3}$ and $E_{5}$ were stopped at their second interim look. The new experimental arms $E_{6}$, $E_{7}$, and $E_{8}$ were added in November 2011, January 2013, and July 2014, respectively. The final stage design parameters of the three added comparisons were similar to those of the original comparisons (i.e. final stage sig. level and power of $\alpha_{i}=0.025$ and $\omega_{i}=0.90$), except that their allocation ratio was set as $A_{6}=A_{7}=A_{8}=1$. Some of the control arm patients recruited from the start of $E_{6}$ and $E_{7}$ are shared between the original family and the new comparisons – see the top section of Figure 1. In all three added comparisons, the final analysis takes place when 267 primary outcome events are observed in the contemporaneously randomised control arm patients.

To calculate the correlation between different test statistics, we needed to estimate (or predict) the shared control arm events of the corresponding pairwise comparisons – Section B of Supplemental Appendix explains this in detail. As the results in Table 1 in Supplemental Appendix show, only seven common control arm primary outcome events were expected to be shared between $E_{7}$ and the original family of pairwise comparisons at their respective primary analysis. For the $E_{6}$ comparison, the (expected) number of common control arm primary outcome events is 77, but it is still a small fraction of the total events required at its main analysis. As a result, the correlations between the corresponding test statistics are quite low in both cases, that is, ${\hat{\rho }}_{k6}^{*}=0.12$ and ${\hat{\rho }}_{k7}^{*}=0.01$, k=1,2,4. Between $E_{6}$ and $E_{7}$ comparisons, the number of shared primary events was expected to be higher ($e_{0,12}=92$) which will result in a slightly higher correlation. But, even in this case, the correlation is well below 0.30. Not only do the first three added comparisons pose distinct research questions, the correlation between the test statistics of the corresponding pairwise comparisons are very low. If the strong control of the FWER was required for the three added arms, the simple Bonferroni correction could have been used to approximate Dunnett’s correction since both the correlation and the amount of overlap between the three comparisons were very low.

### Design application: RAMPART trial

Renal Adjuvant MultiPle Arm Randomised Trial (RAMPART) is an international phase III MAMS trial of adjuvant therapy in patients with resected primary renal cell carcinoma (RCC) at high or intermediate risk of relapse. The control arm (*C*), that is, active monitoring, and the first two experimental arms ($E_{1}$ and $E_{2}$), are due to start recruitment at the same time, with another experimental treatment ($E_{3}$) – which is in early-phase development – expected to be added at least 2 years after the start of the first three arms. The deferred experimental arm, $E_{3}$, will share some of the control arm patients with the other two comparisons and only be compared against those recruited contemporaneously to the control arm over the same period. No head-to-head comparison of the experimental arms is planned, and all the stopping boundaries are prespecified. The trial design has passed both scientific and regulatory reviews, obtained approval from both the EMA and FDA, and started in mid-2018. During reviews of the design, it was deemed necessary to control the FWER at 2.5% (one-sided) in this trial, whether or not the deferred arm is added. Section C of Supplemental Appendix presents a table summary of the design for RAMPART – see https://www.rampart-trial.org/ for full details of the design and trial protocol.

We carried out simulations to investigate the impact of the timing of adding the third experimental arm on the FWER. This was done at 2 years, 3 years, and 4 years into the two original comparisons. The simulation results confirmed our findings that the timing of the addition of $E_{3}$ has no practical impact on the value of FWER. Therefore, the overall (one-sided) type I error rate was proportionally split between the two comparisons that start at the same time and the deferred comparison, that is, $E_{3}$ versus *C*, using the Dunnett correction. Note that in the two comparisons that start at the same time, there is a large proportion of shared control arm information. To make use of the induced correlation between the test statistics of these comparisons, simulations were used to approximate Dunnett probability in this case. Simulations showed that the final stage significance level of 0.0097 in all pairwise comparisons controls the overall FWER at 2.5% when the deferred arm is added later on. Our simulations also showed that the final stage significance level of the two original pairwise comparisons can be increased to 0.015 if $E_{3}$ is not added to buy back the unspent type I error of the third pairwise comparison. This will decrease the required effective sample size, that is, events, in these two comparisons – see the last two columns of Table 2 in Supplemental Appendix – and will bring forward the (expected) timing of the final analysis in both the $E_{1}$ versus *C* (~10 months) and $E_{2}$ versus *C* (~4 months) comparisons.

## Strong control of FWER when required

Opinions differ as to whether the FWER needs to be strongly controlled in all multi-arm trials.^9,6,18–21^ In our view, there are cases, such as examining different doses of the same drug, where the control of the FWER might be necessary to avoid offering a specific therapy an unfair advantage of showing a beneficial effect. However, in many multi-arm trials where the research treatments in the existing and added comparisons are quite different from each other, we would argue that greater focus should be on controlling each pairwise error rate.^9,22^ To support this view, consider the following: if two distinct experimental treatments are compared to a current standard in independent trials, it is accepted that there is no requirement for multiple testing adjustment.^18^ Therefore, it seems fallacious to impose an unfair penalty if these two hypotheses are instead assessed within the same protocol where both hypotheses are powered separately and appropriately. This is seen most clearly when the data remain entirely independent, for example, when these are non-overlapping with effectively separate control groups. Moreover, the statistical reasoning behind the multiplicity adjustment is to limit the possibility of chance as the cause of significant finding. As Proschan and Waclawiw^19^ point out, this becomes less compelling if each comparison answers a different scientific question. In Wason’s 2014 review, this seems to be an emerging consensus among the broader scientific community (see Figure 2 in Wason et al.^6^ review).

**Figure 2.**
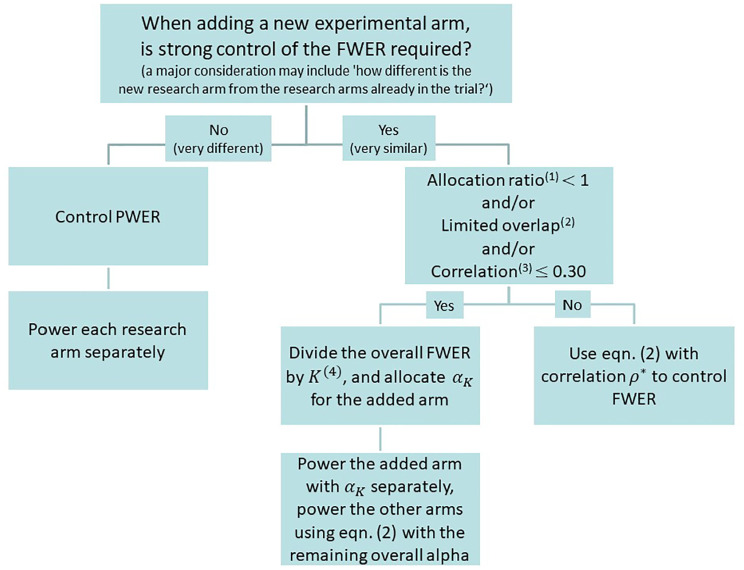
Strategies to control type I error rate when adding new experimental arms. Key: (1) allocation ratio for either of the new or ongoing comparisons; (2) for example, <60% of information time when A=1; (3) correlation between the test statistics of pairwise comparisons; (4) *K* is the total number of pairwise comparisons, including the added arms.

The timing of adding a new research arm can be considered to be a design parameter. Therefore, like any other design parameter, its prespecification is a pre-requisite to the exact calculation of the overall type I error rate. The prespecification of the timing enables the exact calculation of the correlation structure, that is, from formulas in Table 1– which is then used by the Dunnett method to compute the overall type I and II error rates.

However, the findings from our simulations and analytical derivations indicate that by assuming no overlap between the new research arm and that of the existing comparisons, we can relax the requirement for the prespecification of the timing with minimal impact on the FWER. The ‘no-overlap’ condition is when the FWER reaches its upper bound and is strongly controlled regardless of the timing of the addition of the new research arm. In the following, we describe how both the original comparisons and the added arm can be powered using this approach in both planned and unplanned scenarios.

Our results indicate that the timing of adding a new experimental arm to an ongoing multi-arm trial – where the allocation ratio is often one or less, that is, more patients are recruited to the control arm – is almost irrelevant in terms of changing the value of the FWER. Even in cases where there is an overlap (in terms of ‘information time’) of 60%, the impact on the increase of FWER can be negligible. The practical implication of this finding is that in cases where strong control of the FWER is required, one can simply divide the overall FWER by the total number of pairwise comparisons *K*, including the added arms, and take the worst-case scenario of complete independence and design the deferred arm with $\alpha_{K}$ as an independent trial. In this case, they can be considered as separate trials and the new hypothesis can be powered separately and appropriately. If the FWER for the protocol as a whole is required to be controlled at a certain level, as in the RAMPART trial, then the overall type I error can be split accordingly between the original and added comparisons. This seems to be a practical strategy to control the FWER because in most cases the exact timing of the availability of a new experimental therapy may not be determined in advance. If the new experimental arm is not actually added, the final stage significance level of the original comparisons can be relaxed to achieve the target FWER. There might be situations where more experimental therapies are available later than planned at the design stage, that is, the unplanned scenario. In this case, one way to control the FWER for the new set of pairwise comparisons is to reduce the final significance level for the existing comparisons. But, this would increase the (effective) sample size of the existing comparisons and thus the overlap between the new and existing comparisons would increase – which in turn would affect FWER. In this case, a recursive procedure would be required to achieve the desired level for the FWER.

Finally, we emphasise that the decision to control the PWER or the FWER (for a set of pairwise comparisons) depends on the type of research questions being posed and whether they are related in some way, for example, testing different doses or duration of the same therapy in which case the control of the FWER may be required. These are mainly practical considerations and should be determined on a case-by-case basis in the light of the rationale for the hypothesis being tested and the aims of the protocol for the trial. Once a decision has been made to strongly control (or not) the FWER, Figure 2 summarises our guidelines on how to power the added comparison to guarantee strong control of the FWER. We believe this is a logical and coherent way to assess the control of type I error in most scenarios.

## Discussion

It is practically advantageous to add new experimental arms to an existing trial since it not only prevents the often lengthy process of initiating a new trial but also it helps to avoid competing trials being conducted.^1,2^ It also speeds up the evaluation of newly emerging therapies and can reduce costs and numbers of patients required.^3,4^ In this article, we studied the familywise type I error rate and power when new experimental arms are added to an ongoing trial.

Our results show that under the design conditions, the correlation between the test statistics of pairwise comparisons is affected by the allocation ratio and the number of common control arm shared observations in continuous and binary outcomes and primary outcome events in trials with survival outcomes. The correlation decreases if more individuals are proportionately allocated to the control arm. This correlation increases as the proportion of shared control arm information increases, and it reaches its maximum when the number of observations (in continuous and binary outcomes) or events (in survival outcomes) is the same in both pairwise comparisons. Our results also showed that the correlation between the pairwise test statistics and the FWER are inversely related. The higher the correlation, the lower the FWER.

We reiterate that in a platform protocol, the emphasis of the design should be on the control of the PWER if distinct research questions are posed in each pairwise comparison, particularly when there is little or no overlap between the comparisons. To support this, we would argue that the scientific community at large is increasingly judging the effects of treatments using meta-analysis rather than focusing on specific individual trial results.^23^ For this purpose, the readers and reviewers are not concerned about the value of type I error for each trial or a set of such trials.

Another relevant question in a multi-arm platform protocol is what constitutes a *family* of pairwise comparisons. Even in the multi-arm parallel group trials, as Miller^24^ indicated: ‘*There is no hard-and-fast rules for where the family lines should be drawn* .’… In platform trials, this is even more complicated. The difficulty in specifying a *family* arises mainly due to the dynamic nature of a platform trial, that is, stopping of accrual to experimental treatments for lack-of-benefit and/or adding new treatments to be tested during the course of the trial. The definition of a *family* in this context involves a subtle balancing of both practical and statistical considerations. The practical and non-statistical considerations can be more complex in nature, hence the need for (case-by-case) assessment. However, we (and many others) believe the most important criteria is the relatedness of the research questions.^6,19,22^ A consideration that can help to decide the relatedness of the research questions may include ‘how different is the target population for the added arm?’ Moreover, therapies that emerge over time are more likely to be distinct rather than related, for example, different drugs entirely rather than doses of the same therapy. For this reason, each hypothesis is more likely to inform a different claim of effectiveness of previously tested agents. An example is the STAMPEDE platform trial where distinct hypotheses were being tested in each of the new experimental arms, and these do not contribute towards the same claim of effectiveness for an individual drug. In this case, the chance of a false-positive outcome for either claim of effectiveness is not increased by the presence of the other hypothesis.

Although we have focused on single-stage designs, our approach can easily be extended to the multi-stage setting where the stopping boundaries are prespecified. As we have shown in RAMPART, if there are interim stages in each pairwise comparison, the correlation between the test statistics of different pairwise comparisons at interim stages also contribute to the overall correlation structure. Similar correlation formula to those presented in Table 1 can be analytically derived, see Supplemental Appendix, to calculate the interim stages correlation structure. Our experience has shown that even in this case the correlation between the final stage test statistics principally drives the FWER. Our empirical investigation has indicated that even large changes in the correlation between the interim stage test statistics have minimal impact on the estimates of the FWER. Nonetheless, if researchers wish to have the flexibility of non-binding stopping guidelines, then the correlation structure can be estimated in the same manner as discussed in this article by considering the correlation between the final-stage test statistics only. In addition, in the multi-stage designs, there is a chance that some of the original arms are stopped for lack-of-benefit. Even in this case, the FWER is strongly controlled at the prespecified level if the decision to add a new research arm is taken at the initial design stage, that is, planned scenario. However, in the unplanned scenario, the only way to control the FWER for the new set of pairwise comparisons is to suitably reduce the pairwise type I error rates for all the ongoing comparisons, that is, decrease their final significance level, due to the addition of the new research arm.

Furthermore, in our simulations, one experimental arm started with control at the beginning of the trial since the aim was to investigate the impact of the timing of adding new experimental arms on the correlation structure and the value of the FWER. In many scenarios such as RAMPART and STAMPEDE, more than one experimental arms start at the same time in which case there will be substantial overlap in information between the pairwise comparison of these arms to control. If strong control of the FWER is required in this case, Dunnett’s correction (i.e. equation (2)) should be used to calculate the proportion of the type I error rate that is allocated to each of these comparisons as we have done in the case of RAMPART.

Moreover, in some designs such as RAMPART, it is required to control the FWER at a prespecified level. In general, any unplanned adaptation would affect the FWER of a trial. This includes the unplanned addition of a new experimental arm. It will be possible to (strongly) control the FWER if the addition of new pairwise comparisons is planned at the design stage of an MAMS trial as we have shown in the RAMPART example. In this case, the introduction of a new hypothesis will be completely independent of the results of the existing treatments. In platform protocols in general, it becomes infeasible to control the FWER for all pairwise comparisons as new experimental treatments are added to the existing sets of pairwise comparisons.

In this article, we have investigated one statistical aspect of adding new experimental arms to a platform trial. The operational and trial conduct aspects also require careful consideration, some of which have already been addressed by Sydes et al.^1^ In this article, Sydes et al. put forward a number of useful criteria that can be thought about when considering the rationale for adding any new experimental arm. For example, in the STAMPEDE trial, the decisions to add new research arms have been made independently of the accumulating data from the ongoing comparisons. In other words, the decisions to add new arms have been driven by the need to assess new treatment regimens rather than results from the ongoing comparisons. To achieve this, there should be a mechanism in place to ensure that the committee that makes the decision to add an arm is blind to the accumulating results from the ongoing comparisons. Both statistical and conduct aspects require careful examination to efficiently determine whether and when new experimental arms can be added to an existing platform trial.

## Conclusion

The familywise type I error rate is mainly driven by the number of pairwise comparisons and the corresponding pairwise type I error rates. The timing of adding a new experimental arm to an existing platform protocol can have minimal, if any, impact on the FWER. The simple Bonferroni or Šidák correction can be used to approximate Dunnett’s correction in equation (2) if there is not a substantial overlap between the new comparison and those of the existing ones, or when the correlation between the test statistics of the new comparison and those of the existing comparisons is small, less than, say, 0.30.

## Supplemental Material

Supplemetal_Material – Supplemental material for Adding new experimental arms to randomised clinical trials: Impact on error rates Click here for additional data file. Supplemental material, Supplemetal_Material for Adding new experimental arms to randomised clinical trials: Impact on error rates by Babak Choodari-Oskooei, Daniel J Bratton, Melissa R Gannon, Angela M Meade, Matthew R Sydes and Mahesh KB Parmar in Clinical Trials
