# Supplementary material for: Adding new experimental arms to randomised clinical trials: Impact on error rates
Source: Clin Trials. 2020 Feb 17;17(3):273–84. doi: 10.1177/1740774520904346 (PMC7263043; doi:10.1177/1740774520904346)
Supplement: Supplemetal_Material – Supplemental material for Adding new experimental arms to randomised clinical trials: Impact on error rates [file Supplemetal_Material.pdf]

Section A: Correlation structure when adding a new arm

The calculation of FWER requires the specification of the joint distribution of test statistics Z_k $(k = 1, ..., K)$ under the null hypothesis, where K is the number of pairwise comparisons. This involves approximating a multivariate normal distribution function with correlation matrix Σ. The correlation matrix Σ is symmetrical with all diagonal entries $Cov(Z_k, Z_k) = Var(Z_k) = 1$ and all off-diagonal entries, $Cov(Z_k, Z_{k'})$, equal to the correlation between the test statistics of the pairwise comparison k $(k = 1, ..., K)$ - i.e. comparing experimental arm k with control - and the test statistics of the pairwise comparison k' $(k' = 1, ..., K)$ - i.e. comparing experimental arm k' with control. Note that under the null hypothesis each Z_k is normally distributed with unit variance. Therefore, the correlation between the test statistics of pairwise comparisons are equal to their corresponding covariance, $Cov(Z_k, Z_{k'}) = Corr(Z_k, Z_{k'})$. Follmann *et al.*[1] studied the null joint distribution of test statistics Z_k over time and derived analytical formula for Σ based on information times:

$$Corr(Z_k, Z_{k'}) = \begin{cases} \eta_{kk'} . \sqrt{t_k/t'_{k'}} & k \neq k' \\ \sqrt{t_k/t'_{k'}} & k = k' \end{cases} \qquad (1)$$

where t and t' are the information times as defined before $(0 \leqslant t < t' \leqslant 1)$ and

$$\eta_{kk'} = \frac{1}{\pi_0\sqrt{1/\pi_0 + 1/\pi_k}\sqrt{1/\pi_0 + 1/\pi_{k'}}} \qquad (2)$$

where π_0, π_k and $\pi_{k'}$ are the probability of assigning subjects to control and the experimental arms k and k'. If the allocation ratio to all experimental arms is the same $(A_k = A)$, then

$$\eta_{kk'} = \frac{A}{A+1}.$$

As an example, we computed π_0, π_k, η, and $Cov(Z_k, Z_{k'})$ - using eqn. (1) and (2) - for the test statistics of all original pairwise comparisons (including interim stages) in the STAMPEDE trial. Since in the original design of STAMPEDE, $A_k = A = 0.5$ and there were 5 experimental arms, then $\pi_0 = \frac{2}{7}$, $\pi_k = \pi_{k'} = \frac{1}{7}$ $(k = 1, ..., 5)$, and $\eta = 0.33$. In the next section, we derive the analytical formula for $Corr(Z_k, Z_{k'})$. In the survival scenario, we carried out simulations for the STAMPEDE design at the indidual patient data level - results not shown. Our results were identical to those obtained via the trial-level simulation approach developed by Bratton *et al.*[2].

Correlation in survival outcomes

Here, we show how the elements of correlation matrix Σ are estimated when a new arm is added mid-course a trial in trials with survival outcomes. To achieve this, we make use of the asymptotic properties of the log-rank test statistic. Tsiatis[3] showed that over time the sequence of log-rank test statistics approximately has an independent and normally distributed increment structure. We define S_1 and S_2 as the unstandardised log-rank score at times t_1 and t_2 where $t_2 > t_1$. Then approximately,

$S_1 \sim N(\theta V_1, V_1)$, and

$S_2 - S_1 \sim N(\theta(V_2 - V_1), (V_2 - V_1))$

where θ is the log hazard ratio and V_1 and V_2 are the information for θ at times t_1 and t_2.

We can then write down the following Z statistics: Z_1 based on data at time t_1

$Z_1 = S_1/\sqrt{V_1}$

and Z_2 based on the accumulating data between t_1 and t_2:

$Z_2 = (S_2 - S_1)/\sqrt{V_2 - V_1}$

Tsiatis[3] showed that the overall Z statistics for all data at time t_2, i.e. including those in t_1, is:

$$\sqrt{d(t_2)}Z = \sqrt{d(t_1)}Z_1 + \sqrt{d(t_2) - d(t_1)}Z_2 \qquad (3)$$

where $d(t_1)$ and $d(t_2)$ are the number of total primary outcome events at times t_1 and t_2, and Z_2 is the corresponding test statistic of the individuals recruited after information time t_1.

Now, let T be the time of the final analysis, i.e. $t = 1$, and $Z_k(T)$ be the corresponding test statistic for the kth pairwise comparison, i.e. comparison of experimental arm k versus control. Also, let T' be the time and stage of the final analysis for the second comparison to be added later on, and $Z_{(k+1)}(T')$ be the corresponding test statistic at final analysis for the added $(K+1)^{th}$ experimental arm. According to eqn. (3), the log-rank test statistic of the new comparison $Z_{(K+1)}(T')$ at the final analysis can be decomposed into two mutually independent parts: *1*) the log-rank test statistic of the first part where the new comparison and the existing family of comparisons overlap, $Z_{1(K+1)}(t_1)$; and *2*) the log-rank test statistic of the remainder where there is no overlap, i.e. $Z_{2(K+1)}(t_2)$.

Given eqn. (3),

$$Z_{(K+1)}(T') = \sqrt{\frac{d_{1(K+1)}}{d_{(K+1)}}}Z_{1(K+1)}(t_1) + \sqrt{\frac{d_{2(K+1)}}{d_{(K+1)}}}Z_{2(K+1)}(t_2)$$

where $d_{(K+1)}$ is the total number of events in comparison $K+1$ at T', and $d_{1(K+1)}$ and $d_{2(K+1)}$ are the total number of events in part 1 and part 2 (excluding those occurred in Part 1), respectively - i.e. $d_{(K+1)} = d_{1(K+1)} + d_{2(K+1)}$.

Under the proportional hazard (PH) assumption

$$\frac{d_{1(K+1)}}{d_{(K+1)}} = \frac{e_{1(K+1)}}{e_{(K+1)}} \quad \& \quad \frac{d_{2(K+1)}}{d_{(K+1)}} = \frac{e_{2(K+1)}}{e_{(K+1)}}$$

where $e_{1(K+1)}$, $e_{2(K+1)}$, and $e_{(K+1)}(= e_{1(K+1)} + e_{2(K+1)})$ are the control arm events in the newly added $(K+1)$ comparison in Part 1, Part 2 and overall control arm events at time T', i.e. the time of final analysis. Therefore,

$Cov(Z_k(T), Z_{(K+1)}(T')) =$

$$
\begin{aligned}
&= Cov(Z_k(T), \sqrt{\frac{e_{1(K+1)}}{e_{(K+1)}}} Z_{1(K+1)}(t_1) + \\
&\sqrt{\frac{e_{2(K+1)}}{e_{(K+1)}}} Z_{2(K+1)}(t_2)) \qquad (4)\\
&= Cov(Z_k(T), \sqrt{\frac{e_{1(K+1)}}{e_{(K+1)}}} Z_{1(K+1)}(t_1)) + \\
&Cov(Z_k(T), \sqrt{\frac{e_{2(K+1)}}{e_{(K+1)}}} Z_{2(K+1)}(t_2)) \qquad (5)\\
&= Cov(Z_k(T), \sqrt{\frac{e_{1(K+1)}}{e_{(K+1)}}} Z_{1(K+1)}(t_1)) + 0.
\end{aligned}
$$

So,

$Cov(Z_k(T), Z_{(K+1)}(T')) =$

$$\sqrt{\frac{e_{1(K+1)}}{e_{(K+1)}}}.Cov(Z_{1(K+1)}(t_1), Z_k(T))$$

where $Cov(Z_{1(K+1)}(t_1), Z_k(T))$ can be obtained from eqn. (1):

$$
\begin{aligned}
Cov(Z_{1(K+1)}(t_1), Z_k(T)) &= \eta_{k(K+1)} \cdot \sqrt{\frac{e_{1(K+1)}/e_{(K+1)}}{e_k/e_k}} \\
&= \eta_{k(K+1)} \cdot \sqrt{\frac{e_{1(K+1)}}{e_{(K+1)}}}.
\end{aligned}
$$

Then,

$$Cov(Z_k(T), Z_{(K+1)}(T')) = \eta_{k(K+1)} \cdot \frac{e_{1(K+1)}}{e_{(K+1)}} \qquad (6)$$

under the design conditions and with equal allocation ratio among all comparisons the total and shared control arm events between the new $K+1$ experimental arm and the k arms ($k = 1, 2, ..., K$) that start together at the begining are the same, i.e. $e_{(1)} = e_{(2)} = ... = e_{(K+1)} = e_0$ and $e_{1(1)} = e_{1(2)} = ... = e_{1(K+1)} = e_{0,k(K+1)}$.

For trials with continuous and binary outcomes, a similar analytical derivation can be obtained since the corresponding test statistics in these scenarios also has an independent and normally distributed increment structures. However, for these outcome measures the proportion of the common control arm shared primary events - as represented by a ratio in the above equation - is replaced by the proportion of common control shared observations - see below the details.

Correlation in binary outcomes

For binary outcome, we show that the Z test statistics of the difference in proportions, i.e. $p_1 - p_0$, has independent and normally distributed increment structure. This means that at information time $t' > t$

$$Z(t') = \sqrt{t}Z(t) + \sqrt{t'-t}Z(t'-t)$$

Then a similar analytical derivation to that of time-to-event outcomes can be used to derive the formula for ρ^*_{12} in designs with binary outcomes.

In trials with binary outcomes, the outcomes of n_0 individuals in the control (C) arm are $X_{10}, X_{20}, ..., X_{n_0 0} \sim Bern(p_0)$ and those of the experimental (E) arm are $X_{11}, X_{21}, ..., X_{n_0 1} \sim Bern(p_1)$ where $Bern(p)$ stands for the Bernoulli distribution with parameter p. Within our formulation of the null and alternative hypothesis, see Methods section of main text, $H_0^1 : p_1 \geq p_0$ is tested against the (one-sided) alternative hypothesis $H_1^1 : p_1 < p_0$. For simplicity, consider the $1:1$ randomisation, i.e. $n_0 = n_1 = n$, with $\overline{p} = \frac{1}{2}(p_0 + p_1)$. In this case, the test statistic is given by

$$
\begin{aligned}
Z &= \frac{\widehat{p}_1 - \widehat{p}_0}{\sqrt{\frac{2\overline{p}(1-\overline{p})}{n}}} = \frac{\frac{\sum_{i=1}^n X_{i1}}{n} - \frac{\sum_{i=1}^n X_{i0}}{n}}{\sqrt{\frac{2\overline{p}(1-\overline{p})}{n}}} \\
&= \frac{\sum_{i=1}^n X_{i1} - \sum_{i=1}^n X_{i0}}{\sqrt{2n\overline{p}(1-\overline{p})}}
\end{aligned}
$$

If t is the information time when there are m ($m < n$) observations in each group, i.e. $t = \frac{m}{n}$, and t' the information time at $t' = \frac{n}{n} = 1$, then the Z test statistic can be decomposed as:

$$
\begin{aligned}
Z(t') &= \frac{\sum_{i=1}^m X_{i1} - \sum_{i=1}^m X_{i0} + \sum_{i=m+1}^n X_{i1} - \sum_{i=m+1}^n X_{i0}}{\sqrt{2n\overline{p}(1-\overline{p})}} \\
&= \sqrt{\frac{m}{n}} \frac{\sum_{i=1}^m X_{i1} - \sum_{i=1}^m X_{i0}}{\sqrt{2m\overline{p}(1-\overline{p})}} + \\
&\sqrt{\frac{n-m}{n}} \frac{\sum_{i=m+1}^n X_{i1} - \sum_{i=m+1}^n X_{i0}}{\sqrt{2(n-m)\overline{p}(1-\overline{p})}} \qquad (7)\\
&= \sqrt{t}Z(t) + \sqrt{t'-t}Z(t'-t).
\end{aligned}
$$

Correlation in continuous outcomes

For continuous outcomes, it has already been shown that the Z test statistics of the difference in means, i.e. $\mu_1 - \mu_0$, has independent and normally distributed increment structure - see the main text, Methods section, and references[1,3]. Then a similar analytical derivation to that of time-to-event outcomes can be used to derive the formula for ρ^*_{12} in designs with continuous outcomes.

Section B: Shared events in STAMPEDE

.

To calculate the correlation between different test statistics, we needed to estimate (or predict) the shared control arm events of the corresponding pairwise comparisons. For the original family, primary survival results have previously been presented and therefore the observed shared control arm events with future pairwise comparisons (6-7) were used to calculate the correlation; there was no overlap with pairwise comparison 8. For the 6th and 7th pairwise comparisons, we used the ARTPEP software[4] firstly to predict when the primary efficacy analysis for each of these new experimental arms will take place. The predictions were done based on the survival function we observe for the control arm of the STAMPEDE trial and the number of control arm events required for that comparison. Then, under similar assumptions, we predicted the number of control arm events likely to be observed at the primary analysis that are shared with other previously added arms. The observed accrual and event rates amongst only those control arm patients shared across the deferred comparisons were used as input to the ARTPEP software. This then outputs the number of control events expected over time for those shared patients. Based on the first predictions, for when each of the deferred arms might report primary results, the ARTPEP output from the shared control arm patients enabled an estimation of the number of shared control arm events at that time.

Section C: RAMPART design

In RAMPART, patients in E_1 receive 1500mg of durvalumab for one year, and patients in E_2 receive a combination therapy of durvalumab and tremelimumab. Disease-free survival (DFS) is the primary outcome used throughout the trial at all analyses. For the sample size calculations, the target hazard ratio (HR) for E_1 vs. C comparison was assumed 0.75. However, a larger effect size of 0.70 is targeted for the combination therapy comparison. As a result, the two pairwise comparisons have different follow-up periods. All pairwise comparisons share some of the control arm events, with the two pairwise comparisons that start at the same time sharing the most, and the deferred comparison sharing the least control arm information. The design has formal looks for both lack-of-benefit and efficacy. (Technical detail on the implementation of the efficacy stopping rules in Royston *et al.* design can be found in articles by Blenkinsop *et al.*[5 6].) Table 2 presents the design parameters for each of the pairwise comparisons, and the total number of control arm events required to trigger the final analysis. For full details of the design, please see the trial protocol at https://www.rampart-trial.org/.

Table 1. *Correlation structure between the newly-added comparisons as well as with those of the original ones in the STAMPEDE trial - see Figure 1 in the main text.*

Expl. arm		E_1, E_2, E_4 exp. arms			E_7 exp. arm			E_8 exp. arm		
	e_0	$n_{0,kk'}$	$e_{0,kk'}$	$\widehat{\rho}^*$	$n_{0,kk'}$	$e_{0,kk'}$	$\widehat{\rho}^*$	$n_{0,kk'}$	$e_{0,kk'}$	$\widehat{\rho}^*$
E_6	267	377	77	0.12	-	-	-	-	-	-
E_7	267	27	7	0.01	269	92	0.17	-	-	-
E_8	267	0	0	0	0	0	0	405	57	0.11

Key: e_0, total control arm primary outcome events required at the final analysis; $k = 1, 2, 4$, $k' = 6, 7, 8$ $n_{0,kk'}$, shared control arm patients; $e_{0,kk'}$, (projected) number of shared primary outcome events in control arm; $\widehat{\rho}^*$, the estimates of correlation between test statistics of pairwise comparisons.

Table 2. *Design parameters for the MAMS RAMPART trial. The FWER is controlled at 0.025 (one-sided) in both scenarios, when E_3 is added later on and when E_3 is not added.*

Comp.	Target	No of	Interim boundaries		Final sig. level (α) [2]		Total control arm events	
	HR	int. stages	lack-of-benefit	efficacy	E_3 added	no E_3	E_3 added	no E_3
E_1 vs. C	0.75	3	$0.0143, 0.062, 0.034$	0.001[1]	0.0097	0.015	416	380
E_2 vs. C	0.70	1	0.05	0.001	0.0097	0.015	276	252
E_3 vs. C	0.75	1	0.30	0.001	0.0097	–	331	–

1) boundaries for all three interim stages; 2) the overall pairwise power is 0.87 in all pairwise comparisons.

References

1. Follmann DA, Proschan MA, Geller NL. Monitoring Pairwise Comparisons in Multi-Armed Clinical Trials. Biometrics. 1994;50(2):325–336.
2. Bratton DJ, Choodari-Oskooei B, Royston P. A menu-driven facility for sample size calculation in multi-arm multi-stage randomised controlled trials with time-to-event outcomes: Update. Stata Journal. 2015;15(2):350–368.
3. Tsiatis AA. The asymptotic joint distribution of the efficient scores test for the proportional hazards model calculated over time. Biometrika. 1981;68:311–315.
4. Royston P, Barthel FMS. Projection of power and events in clinical trials with a time-to-event outcome. The Stata Journal. 2010;10(3):386–394.
5. Blenkinsop A, Parmar MKB, Choodari-Oskooei B. Assessing the impact of efficacy stopping rules on the error rates under the MAMS framework. Clinical Trials. 2018;16(2):132–142.
6. Blenkinsop A, Choodari-Oskooei B. Multiarm, multistage randomized controlled trials with stopping boundaries for efficacy and lack of benefit: An update to nstage. Stata Journal. 2019;19(4):782–802.
